# Integrated Pest Management of Coffee Berry Borer: Strategies from Latin America that Could Be Useful for Coffee Farmers in Hawaii

**DOI:** 10.3390/insects7010006

**Published:** 2016-02-03

**Authors:** Luis F. Aristizábal, Alex E. Bustillo, Steven P. Arthurs

**Affiliations:** 1 Mid-Florida Research & Education Center, University of Florida/IFAS, 2725 Binion Rd, Apopka, FL 32703, USA; spa@ufl.edu; 2 Centro de Investigación en Palma de Aceite (Cenipalma), Calle 20 A No. 43 A-50, Pisos 2 y 4, Bogotá D.C., Colombia; alexe.bustillo@gmail.com

**Keywords:** *Hypothenemus hampei*, cultural practices, biological control, monitoring, post-harvest, *Beauveria bassiana*, *Cephalonomia stephanoderis*, *Prorops nasuta*, *Phymastichus coffea*, *Cathartus quadricollis*

## Abstract

The coffee berry borer (CBB), *Hypothenemus hampei* Ferrari (Coleoptera: Curculionidae: Scolytinae) is the primary arthropod pest of coffee plantations worldwide. Since its detection in Hawaii (September 2010), coffee growers are facing financial losses due to reduced quality of coffee yields. Several control strategies that include cultural practices, biological control agents (parasitoids), chemical and microbial insecticides (entomopathogenic fungi), and a range of post-harvest sanitation practices have been conducted to manage CBB around the world. In addition, sampling methods including the use of alcohol based traps for monitoring CBB populations have been implemented in some coffee producing countries in Latin America. It is currently unclear which combination of CBB control strategies is optimal under economical, environmental, and sociocultural conditions of Hawaii. This review discusses components of an integrated pest management program for CBB. We focus on practical approaches to provide guidance to coffee farmers in Hawaii. Experiences of integrated pest management (IPM) of CBB learned from Latin America over the past 25 years may be relevant for establishing strategies of control that may fit under Hawaiian coffee farmers’ conditions.

## 1. Introduction

Native to Africa, two species of coffee, *Coffea arabica* L. and *C. canephora* Pierre ex A. Froehner (Gentianales: Rubiaceae) are produced on over 10 million ha in 80 countries located in tropical and subtropical regions [1,2,3]. According to ICO [4], coffee production worldwide has increased by over 50% in the past 25 years, from 93 million bags (60 kg) in 1990 to 143 million bags in 2014/15. The total value of the coffee industry during 2012 was estimated at US $173 billion [4]. In Hawaii the area planted under coffee was estimated at 3440 ha with farm revenue US $50.3 million in 2014/15 [5]. Since the coffee berry borer (CBB), *Hypothenemus hampei* Ferrari (Coleoptera: Curculionidae: Scolytinae), the most economically important insect pest in coffee plantations [3,6,7,8,9], was reported in Hawaii in September 2010 [10], coffee farmers from the islands have faced losses in yields and revenue. A similar situation occurred in Brazil in 1913, in Colombia in 1988 and in other Latin American countries in 1970s, 1980s, and 1990s when the CBB was detected [7,11,12,13,14]. Here we summarize lessons learned regarding the integrated pest management (IPM) of CBB from Colombia and Latin America that could be applied to Hawaii and elsewhere.

Three important topics about the CBB discussed in this review are (1) Biology, ecology, and behavior, which are needed to understand the pest and its management; (2) IPM, including components such as monitoring, cultural control, use of mycopesticides, natural enemies, post-harvest control strategies, and coffee rejuvenation; and (3) Recommendations for coffee growers, Extension workers, and IPM researchers.

## 2. Biology, Ecology, and Behavior of CBB

Establishing an IPM program for CBB requires knowledge about its biology, ecology, and behavior within a specific region. The pest is influenced by crop phenology, especially by periods of coffee blooms, development of berries, and harvesting strategies, (e.g., timing applications of mycopesticides with periods of high CBB flight activity, or cultural control practices with harvesting periods). Several authors have reported comprehensive reviews about the CBB biology and control strategies [3,7,8,11,15,16,17,18].

### 2.1. General Biology of CBB

All CBB life stages develop inside coffee berries, which make insecticidal control difficult [7,8,9]. The process of colonization of a healthy berry starts when a mated female initiates the search for new oviposition habitats [9]. Developing green coffee berries with >120 days growth post flowering and >20% dry weight are preferentially selected [8,19]. Younger green berries (≥60 days old) may also be infested by CBB, but the female has to wait in a penetration channel, until the berries achieve >20% dry weight in order to bore a gallery and initiate oviposition [8,19]. Early infestation of green berries (60–90 days old) may result in premature drop from trees [7]. Female CBB may attack developing berries until they become mature (>224 days) at harvest time [8]. In general, a hardened developing berry is bored by the female, then irregular tunnels and galleries are excavated in which the female lays her eggs. Females lay between one and three eggs per day over 20 days [15]. After resting, females repeat the oviposition process. According to Barrera [20] a female may lay up to 119 eggs inside a single berry. The CBB develops through six life stages: egg, larva (first and second instar), pre-pupa, pupa, and adult. The life cycle requires at least 25 days and may take >60 days depending on temperature and hardness of the berry (endosperm) [8]. The founding female does not leave the berry and remains with her progeny [21]. The mating system is functionally haplo-diploid [22], allowing mating between siblings, which have a female-biased sex ratio of around 10:1. Since developing berries typically require 200–250 days from flowering to harvest, individual berries can support multiple generations of CBB [21]. Jaramillo *et al.* [23] reported about 4 generations of CBB per year in Tanzania and Colombia, with 3 generations in Kenya, and 2 in Ethiopia. There is a variation of longevity of CBB adults. Female life-span ranges from 81–282 days [15], and average 131 days [24]. Male are shorter lived with longevity reported at 40 days [15] and 52 days [25]. 

### 2.2. Coffee Plant and CBB

The development of the coffee crop, specifically flowering, development of berries, and harvest time, are related to climate. For example, flowering occurs when rainfall follows a dry period as physiological response of the plant due to water limitation [26,27]. In Colombia, flowering to berry maturation requires 7–9 months depending on temperatures [28]. At 1200 m above sea level (masl) (average temperature 22 °C) berries need 7 months for maturity, at 1400 masl (20.5 °C) 8 months, and at 1750 masl (19 °C) 9 months [28]. In the central coffee region of Colombia, there are two flowering periods (January and February and August and September) that allow two harvest periods (September until November and March until May) [26,28]. There is also some additional flowering that allows developing berries throughout the year. This situation promotes CBB because the beetle finds food and shelter for reproduction all year long, making control more challenging. Coffee berries reach 20% dry weight between 110 and 140 days after blooms [29,30]. At this point berries become more firm (hardness) allowing infestation and reproduction of CBB. Ruiz [31] reported that developing berries > 150 days old (27% dry weight) are attacked by CBB and oviposition started after 4–5 days. Baker [8] showed that CBB females prefer older berries 150–240 days (>20% dry weight) over younger berries < 90 days old. Higher density plantations and older larger trees provide more habitats for CBB and make control more difficult. Highest CBB infestations levels were observed on lower branches in contrast to the middle and top of the tree [32]. According to Bustillo [33], records of flowering periods are necessary to predict harvest time, peak harvest, and critical periods of potential attack by CBB.

### 2.3. Alternative Host Plants

CBB adults have been observed in plants other than the genus *Coffea* [9]. However, the insect life cycle is only known to be completed on coffee species [34]. The presence of CBB adults on other plants may represent transitory shelter until developing coffee berries are available. Plants among the families Fabaceae, Euphorbiaceae, Passifloraceae, Rubiaceae, Arecaceae, Araliaceas, and Anacardiacease were evaluated as potential host plants for CBB in Kona, Hawaii [35]. Some CBB adults were found on “hoale koa” *Leucanea leucocephala* (Lam.) but were unable to use these plants as hosts [35].

### 2.4. Sources and Emergence of CBB

**Figure 1:**
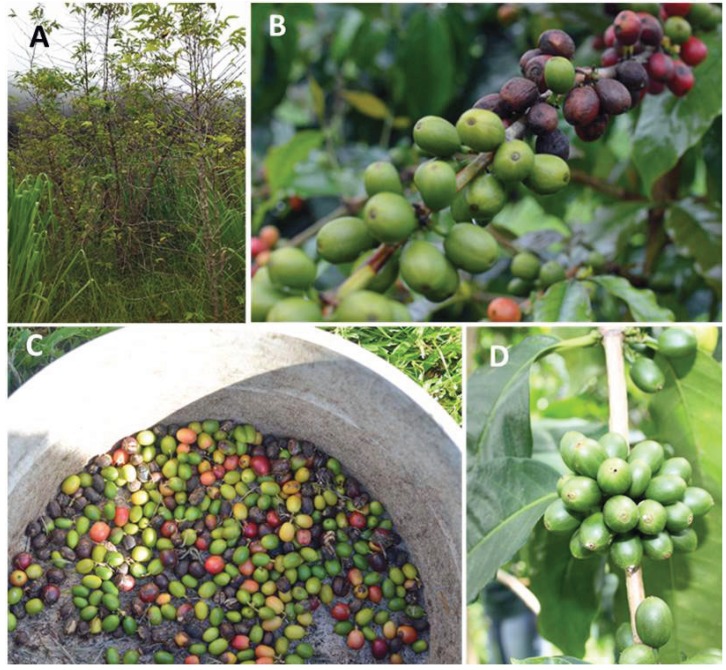
Feral coffee in Hawaii may allow the spread of CBB, (**A**); over-ripe and raisin berries are high source of CBB (**B**); berries collected from the ground helps control CBB (**C**); green berries attacked by CBB (**D**). Photos taken in Pahala, Hawaii by Libia C. Mahé, and Luis F. Aristizábal.

The availability of food and prevailing weather conditions influence the emergence of adult female CBB from infested berries. High relative humidity (>90%) especially after rainfall and increase in temperatures stimulate emergence of the CBB female [8,36,37]. In Colombia the pruning practice known as “zoqueo”, which is the rejuvenation of old coffee trees after a decline in production, is a major source of CBB. After they are stumped, the old trees and fallen infested berries release CBB and become a pest reservoir [8,38]. Similarly, feral coffee plantations (abandoned by farmers) in Kona and Pahala regions in Hawaii provide a reservoir of CBB habitat. High infestation of CBB (81%) on feral coffee plantations may contribute to the spread of CBB in Hawaii (Table 1, Figure 1A, farm 11* in Pahala (Infestation of CBB and degree of penetration of CBB inside berries was conducted according to thirty tree sampling method developed by Bustillo *et al.* [7] and recommended in Hawaii by Kawabata *et al.* [39]. The evaluation was conducted by coffee farmers and the first author. See details of methodology on Section 3.1 Sampling CBB Populations)). Ripe and over-ripe berries that are left on the trees after harvest and those that fall on the ground serve as a source of new CBB infestations (Figure 1B,C). During dry conditions, infested fallen berries can contribute a large number of CBB females that are stimulated to emerge by high relative humidity (>90%) after rainfall [8].

**Table 1:** Infestation levels of coffee berry borer (CBB), positions of the CBB (AB-CD), infection by *B. bassiana* (Bb), and mortality of CBB on coffee farms from Pahala, and Kona, Hawaii in September 2015.

Farm	CBB	Bb	AB	CD	Mortality	Empty
1	66.4	64.5	32.7	16.3	39.4	11.4
2	49.9	42.3	18.8	15.9	44.9	20.2
3	26.7	16.4	23.8	6.8	30.6	38.6
4	23.1	61.9	18.0	19.4	47.2	15.4
5	14.7	23.8	24.4	13.9	15.1	46.5
6	10.2	19.7	9.4	26.4	30.1	33.9
7	4.2	27.5	36.3	26.3	8.0	19.0
8	3.3	11.0	64.8	17.8	1.9	18.2
9	3.2	9.3	39.3	45.4	6.0	9.0
10	2.9	22.0	10.0	8.0	26.0	56.0
Average ± SEM	20.5 ± 6	29.8 ± 6	27.8 ± 5	20.3 ± 3	24.9 ± 4	26.8 ± 4
11 *	81.0	44.0	18.8	13.5	52.7	18.9
12 **	0.7	9.0	n/a	n/a	n/a	n/a

**Notes:** CBB = % Infestation of CBB, Bb = % CBB infected by *B. bassiana*, AB and CD = % of CBB penetration inside berry (AB and CD positions), Mortality = % Dead CBB female, Empty = % berries infested but without CBB female. 11 * = Feral coffee plantation abandoned in Pahala, Hawaii. 12 ** = New coffee plantation rejuvenate by stump pruning in a block, Greenwell coffee farm in Kona, Hawaii.

### 2.5. Visual and Olfactory Attractants of CBB

The response of female CBB to visual cues such as color and movement was reported by several authors [40,41,42,43]. The beetles showed preference to black and red in studies conducted with natural and artificially colored berries [40,41,42,44]. The female CBB has larger eyes and responds to movement while males have rudimentary eyes and poor vision [43,45]. Females are also attracted to kairomones released by developing berries [42,44,46,47]. Coffee berry kairomones are composed of different alcohols [48]. The use of visual and olfactory attractants for CBB is used to improve monitoring through the use of lures. For example, the discovery of CBB females response to methanol and ethanol allowed the application of alcohol based traps for monitoring CBB populations under field conditions in Mexico [49], Central America [50,51], Brazil [52,53], Hawaii [35] and Colombia [54].

### 2.6. Flight and Dispersal of CBB

The flight of CBB females was reported by Baker [11]. Laboratory tests showed that CBB can fly unaided for at least 22 min and 100 min when tethered. The flight activity peak was observed at 2:00 pm [25] and between 1:30 and 3:30 pm [50]. Males have degenerate wings and cannot fly [9]. According to Decazy [16], most CBB fly short distances, but some disperse long distances searching for berries. Baker [8] reported that CBB from a “zoqueo” coffee plot immigrated to coffee plantation at least 500 m distant. Long distance dispersal may be achieved via wind, animals, and humans, with international dispersal occurring inadvertently via the coffee trade [34].

### 2.7. Spatial Distribution of CBB

Initial infestations of CBB in coffee plantations assume an aggregated distribution [8,20]. Initial colonizing CBB produce odors that attract other females explaining the aggregation behavior [44]. CBB infestations (hot spots) are most common in areas with elevated shade or humidity [20]. In Colombia, CBB “hot spots” are observed in the edges (first three rows) and depressions or lower areas of plantations older than 3 years [33]. As the population increases, the spatial distribution of CBB becomes more regular. In Guatemala, infestation levels >10% showed a non-aggregated spatial distribution [55]. In general, high risk areas for CBB infestation occur adjacent to facilities where the harvested coffee is processed and near roads close to buildings and neighbor coffee plantations. Therefore, monitoring efforts should be intensified in these locations, especially during harvest when berries are moved.

### 2.8. Effect of Seasonal Rainfall 

During the rainy season, CBB populations are often reduced since wet conditions promote natural mortality factors. For example, fallen berries decompose faster thus limiting the period for emergence [56]. Wet and humid conditions promote entomopathogenic fungi such as *Beauveria bassiana* (Balsamo-Crivelli) Vuillemin, *Metarhizium brunneum* (Petch), *Isaria fumosorosea* (Wize), and *Lecanicillium lecanii* (Zimm.) (Viégas) which infect CBB [3,7,8,57]. Dry seasons promote the reproduction of CBB on fallen berries or left on the trees. However, a prolonged dry season may reduce CBB populations, since the beetle is sensitive to berry desiccation [8,36]. In Hawaii coffee is planted at different elevations. Plantations at lower elevation during the dry season may see higher reproduction and dispersal of CBB, making its control more challenging. In Pahala and Hilo, precipitation and the number of cloudy days is higher than in the Kona coffee region, which may improve the use of *B. bassiana* against CBB.

### 2.9. Effect of Shade on CBB

Several authors reported highest CBB infestations in plantations grown under shade [58,59,60,61,62]. According to Vega *et al.* [3], the CBB evolved in the shade forest in Africa and the insect is better adapted to environments with associated high humidity. However, coffee plantations grown with shade trees have an advantage in that *B. bassiana* may work better as an insecticide in those conditions. Vega *et al.* [3] report additional benefits provided by shade trees, including prevention of soil erosion, higher biodiversity, improvement in organic matter, and reduced temperature. In addition, shade trees are a requirement for organic coffee production and support the biodiversity of natural enemies of CBB such as birds, predators (ants and beetles), and parasitoids. For example, coffee plantations located in Captain Cook and South Kona, Hawaii, are grown with macadamia trees, *Macadamia* spp. F. Muell (Proteales: Proteacea) [63].

## 3. Strategies on Integrated Pest Management

Components of IPM for CBB include sampling and monitoring, cultural harvesting, use of *B. bassiana*, post-harvest control, release of parasitoids, and pest management during “zoqueo”. Many studies report the effectiveness of individual management components for CBB from different regions [3,7,8,9,17,18,51,64,65,66]. However, few report the effectiveness of multiple components applied as an IPM program [7,33,67,68]. Moreover, from 1865 publications of CBB [69], few reports compare the costs of different management strategies for CBB, [7,8,68,70,71,72,73].

The goal of coffee farmers is the production of high quality coffee at the best market price produced at lowest cost. In Hawaii, most coffee is sold as “specialty” coffee, which commands a price premium. However, the CBB reduces quality and price, causing loses of yields, and increased costs [74]. According to Shriner [75], before CBB arrived to Hawaii (2010), “Extra Fancy” comprised 25% of the Kona crop. However, from 2011–2013, no coffee was certified above “Prime” (the lowest grade) due to CBB defects. The loss of “Extra Fancy”, as well as “Fancy” and “Number One” grades was a huge economic blow to the industry’s export market [75]. The current levels of coffee infestation as measured by percent CBB infestation in Hawaii in 2014 was 15% [75] and averaged 20% in our 2015 survey (Table 1) which indicates the severity of the CBB problem. There are several tools to control the CBB; however, history shows that the successful establishment of an IPM program requires significant implementation [7,8,68,76]. An IPM program combines control strategies based on regional phenology and cost/benefit analysis of specific tactics [8,77]. 

### 3.1. Sampling CBB Populations

**Figure 2:**
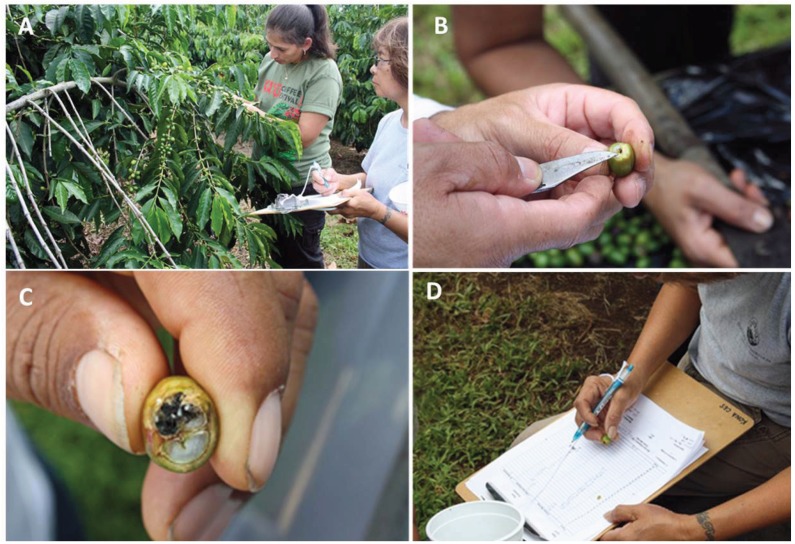
Evaluating the infestation levels of CBB (**A**), in position AB the CBB is vulnerable to insecticides (**B**); in the CD position CBB only can be controlled by removal of berries or natural enemies (**C**); recording the information (**D**). Photos taken in Pahala, Hawaii by Juan A. Aristizábal and Luis F. Aristizábal.

An important aspect of an IPM program for CBB is determining the location and infestations levels of the pest, and the degree of berry penetration (Figure 2). According to Bustillo *et al.* [7], random sampling is the most appropriate method to estimate CBB populations. Several sampling methods for CBB are reported [7,8,78,79,80,81,82,83]. The thirty tree sampling procedure known as “Cenicafé method” in Colombia [7] was introduced in Hawaii by Luis F. Aristizábal in 2012 [39] (Figure 2A). A brief description of the sampling method follows (1) divide the coffee farm into independent lots according to plantation age; (2) randomly select 30 per 5000 trees; (3) select a representative branch in the middle of the tree containing 30–100 developing berries; (4) examine all green berries for CBB entry hole; (5) record the number of green berries and those with CBB hole; (6) repeat the process moving in a zig–zig pattern through the plantation; (7) calculate percent CBB infestation for each lot.

In addition to calculating infestation severity, the degree of penetration of CBB inside the berry should be evaluated, since this determines the likelihood that insecticides will be effective. For this, 100 infested green berries are collected throughout each coffee lot. A CBB in the AB position indicates that the female beetle initiated penetration but not reached the endosperm. Therefore no oviposition or immature life stages will occur in the berry (Figure 2B). In the AB position, the female is relatively exposed and vulnerable to applications of insecticides, as well as natural enemies (predators and parasitoids). By contrast, a CBB in CD position has entered the endosperm and initiated reproduction (Figure 2C). In the CD position the CBB and its progeny are protected from insecticides and the only strategies for control are cultural collection of the berries before the CBB progeny emerge or through the establishment of natural enemies (predators and parasitoids). According to Bustillo *et al.* [7] the thirty tree method allows the identification of “hot spots” in the coffee plantation, which is critical. Application of insecticides (such as *B. bassiana*), releases of parasitoids and predators or targeted collection of ripe, over-ripe, and raisin berries from trees and the ground should focus on “hot spots”.

**Figure 3:**
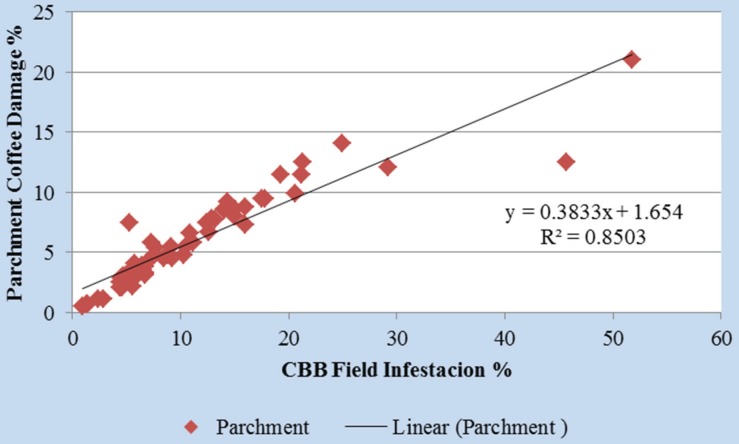
Correlation between CBB infestations levels and damage on parchment coffee. Data from 30 coffee farms in Quindío, Colombia (Source: Aristizábal [84]).

In Colombia, the criteria to spray insecticides is >2% infestation with >50% of CBB in AB position applied within 5 days of monitoring [7,68]. In a recent study conducted in over 80 coffee lots, monthly sampling using the 30-tree method and targeted control measures using the AB/CD criteria resulted in dramatically reduced CBB infestations, while reducing the costs, and increasing the proportion of premium-grown coffee [68]. When executed properly, the 30-tree sample method correlates CBB infestations levels in field and parchment coffee (Figure 3). The localization of pest “hot spots”, and the degree of penetration inside berries (positions AB–CD) helps farmers make decisions about control strategies and evaluate the effectiveness of IPM programs. 

### 3.2. Monitoring CBB with Alcohol Traps 

Alcohol traps have been used to monitor the seasonal flight activity of CBB in Central and South America [49,50,51,52,53,54,85]. The lure is typically enclosed in a semi-permeable plastic membrane that allows release of alcohols at an optimal rate of 186 mg/day [50]. The effectiveness of traps is optimized by color (red or white), the mixture of alcohols (ethanol: methanol; in a 3:1 or 1:1 ratio), and location (0.5–1.5 m high). However trap efficiency is influenced by other factors, including weather conditions, and pest infestation levels [35,50,52,86,87,88,89,90]. 

Traps are used to estimate periods of CBB dispersal. In Brazil, highest CBB activity was observed during the postharvest period (August to February), with peaks during October and November [85]. In the central coffee region of Colombia, highest flight activity was observed January to March [54]. Capture rates up to 6120 CBB per trap/week were observed in farms with high infestation (17%–28% of berries) [54]. In Hawaii, peak CBB activity was observed in November (south Kona) and January (central Kona), during the peak and late harvest periods respectively [35]. Messing [35] reported that plastic pouch with mixture of alcohols were more effective baits compared with open vials of alcohols. Through early detection of seasonal flight activity, coffee farmers can make better control decisions by applications of *B. bassiana* with high activity of CBB. 

### 3.3. Cultural Control Practices 

**Figure 4:**
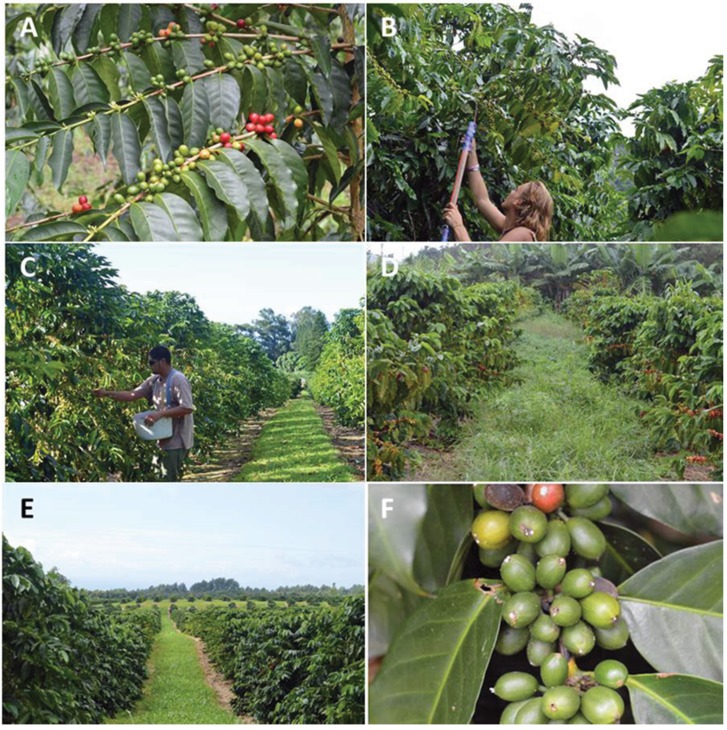
Coffee tree with berries at developmental stages from flowering to maturity (**A**); tall trees with many verticals and narrow spacing (<2 m) makes collection difficult (**B**); trees with <4 verticals and spacing >2 m simplifies harvest (**C**); weeds obscure fallen berries (**D**); weed management facilitate harvesting (**E**); CBB in AB position infected by *B. bassiana* (**F**); Photos taken in Pahala, Hawaii, by Libia C. Mahé, and Luis F. Aristizábal.

Before CBB was detected in Colombia in 1988, about 10% of coffee berries were left on trees and the ground after harvesting [91]. Coffee farmers did not consider this loss significant. However, after CBB was detected, this situation changed, since the residual coffee berries became the main reservoir of CBB that infested new coffee berry cycles [7]. Under Colombian conditions, where several flowering cycles occurs during the year, but only two harvest periods, the control of CBB was challenging. Since all CBB life stages occur inside a berry, removing infested berries became the goal. Regular harvesting of ripe, over-ripe, and raisin (dried) berries during harvest periods reduced CBB from 70% to <6% infested berries [92]. The practice of frequent (2–3 week intervals) and more efficient harvesting has become a primary tool to manage CBB in Colombia [7,67,73,93,94,95]. A similar situation of continuous flowering/production cycles of coffee was observed in Pahala and elevated coffee plantations in Kona, Hawaii (Figure 4A). Therefore, increased frequency and efficacy of harvesting mature, over-ripe, and raisin berries is a strategy that needs to be evaluated under Hawaiian conditions. Since labor (harvest workers) is more expensive in Hawaii compared with Colombia and other countries in Latin America, the cost/benefit aspects of cultural harvesting *versus* other control strategies needs to be evaluated. 

The success of cultural harvesting depends on how efficiently ripe, over-ripe and raisin berries are collected [7,67]. According to Bustillo *et al.* [7], the goal is <5 such berries per tree, with >10 berries resulting in an ineffective practice, while 6–10 berries/tree means that cultural control is partially effective. Working with coffee farmers through a participatory research program, Aristizábal *et al.* [73,76,95] reported significant control of CBB over two years though effective harvesting alone. In another example, the control of CBB on an 110 ha specialty coffee farm in Colombia was significantly enhanced through training harvest workers to improve their cultural harvesting effectiveness. Results showed that trained workers left an average of 6.5 berries per tree postharvest, down from 22.5 berries per tree, resulting in 0.7% CBB damage on parchment coffee, down from 2.3% prior to training [68].

Similar reports have emerged from other countries where cultural harvesting (re-picking) is implemented. In Brazil [96] and Guatemala [70], improved CBB control occurred when re-picking after harvest was conducted. In Mexico, the re-picking practice was as effective as two applications of insecticides in the control of CBB [71]. 

In Hawaii, most coffee pickers are Hispanic or Micronesian (Pacific Islander) and typically paid high wages (US $0.60/pound or higher during the main season and hourly US $12–15 at other off-peak times [75]. Further, few coffee farmers train pickers according to the criteria outlined above. For example, between 20 and 45 berries left per tree and >23 berries on the ground per tree were observed after a round of harvest in coffee farms in Pahala and Kona, Hawaii [63]. Under this scenario control of CBB would be difficult even if insecticides were applied frequently. Training workers to collect old berries, and prevent fallen berries, is needed in Hawaii. However, training pickers may be a challenge due to a shortage of laborers and difficulty retaining them. There is also a language barrier, primarily for Micronesian laborers [75], for those reasons, farmers may have difficulty enforcing standards. 

In Hawaii, a “sanitation” involving the total removal of berries at the end of harvest season [39], may not apply for some regions such as Pahala, Kona and other elevated plantations due to continuous flowering and berry presence all year [63]. In some cases, coffee farmers may wait until infested developing berries become mature for harvesting. Since CBB generally only infests one of the two endosperm seeds (coffee berry), farmers can save 50% of an infested berry. In addition, there are more life stages of CBB on a raisin (46.3 individual per berry), over-ripe (23.4), and mature (9.1) berries than in a green berry (2.9) [7]. This means that over-ripe and raisin berries should be preferentially collected during harvesting. Therefore, coffee farmers and pickers should understand that collecting mature, over-ripe, and raisin berries and preventing berries falling during harvest will control CBB populations. Other aspects that will influence the effectiveness of harvesting and insecticidal control include (1) the distribution and density of the coffee trees; (2) the number of vertical branches (shoot growth) per tree (Figure 4B,C); (3) weed control which enhances collection of berries on the ground (Figure 4D,E) and (4) optimal fertilization to promote high production and quality coffee.

### 3.4. Use of B. bassiana 

The entomopathogenic fungus *Beauveria bassiana* naturally infects CBB in countries including Brazil [97,98] Ecuador [56], Colombia [99], Honduras [100], Mexico [101], Costa Rica [102], and Puerto Rico [103]. The natural occurrence of *B. bassiana* infecting CBB is variable, and has been reported at <1% beetles in Brazil [104], but higher in other regions, *i.e.*, 44% in Nicaragua [105], 60% in India [106] and reached 71% in a study in Cameroon [107]. The fungus has been developed as an environmentally safe bioinsecticide that is sprayed against CBB but is not toxic to workers and has low impact on non-target organisms including CBB natural enemies. The effectiveness of *B. bassiana* under field conditions depends of several factors, including the strain, concentration, virulence, weather conditions, and application efficiency [7,8,108,109,110,111]. Previous reports document CBB sprayed with *B. bassiana* under field conditions in Colombia achieved infection rates of 69% [112], 91% [113], 64% [114] and 67% [115]. Cruz *et al.* [116] stated that mixtures of *B. bassiana* strains may result in synergism against CBB. Spraying *B. bassiana* is another strategy to control CBB in fallen berries [117,118,119]. Using this approach, the CBB population was reduced by 75% on fallen berries [114]. Recent research has focused on the use of *B. bassiana* as a fungal endophyte (colonizing live coffee plants) [120,121,122]. Although *B. bassiana* was confirmed as a viable endophyte in inoculated plants, its establishments was relatively short lived (<6 months) [121]. 

According to Hollingsworth [123], in Hawaii, there is concern about native insects. Initially *B. bassiana* was classified as a “restricted” microorganism in the Islands, meaning that it could be initially imported under permit for research, but not used commercially. In February 2011, based on the need and safety assessment, the Hawaii Board of Agriculture permitted products containing the GHA strain to be imported and used by growers [123]. There is currently a voluntary federal subsidy program for growers to apply *B. bassiana* in plantations infested by CBB [75]. According to Shriner [75], currently, close to 4700 acres (out of an estimated 6000) have contracted to the subsidy program. Participating growers are required to monitor using the 30-tree method before receiving the *B. bassiana* product, and instructed how to apply to optimize its effectiveness [75]. Early field trials with commercial products showed infections between 25%–45% of founder CBB females on green berries during peak fight activity [124]. The author’s survey of different farms showed variable infection rates (9%–67%) (Table 1; Figure 4F). Improvements in the use of *B. bassiana* as a biopesticide and potential endophyte are warranted to increase its effectiveness under Hawaiian conditions. 

### 3.5. Post-Harvest Control

**Figure 5:**
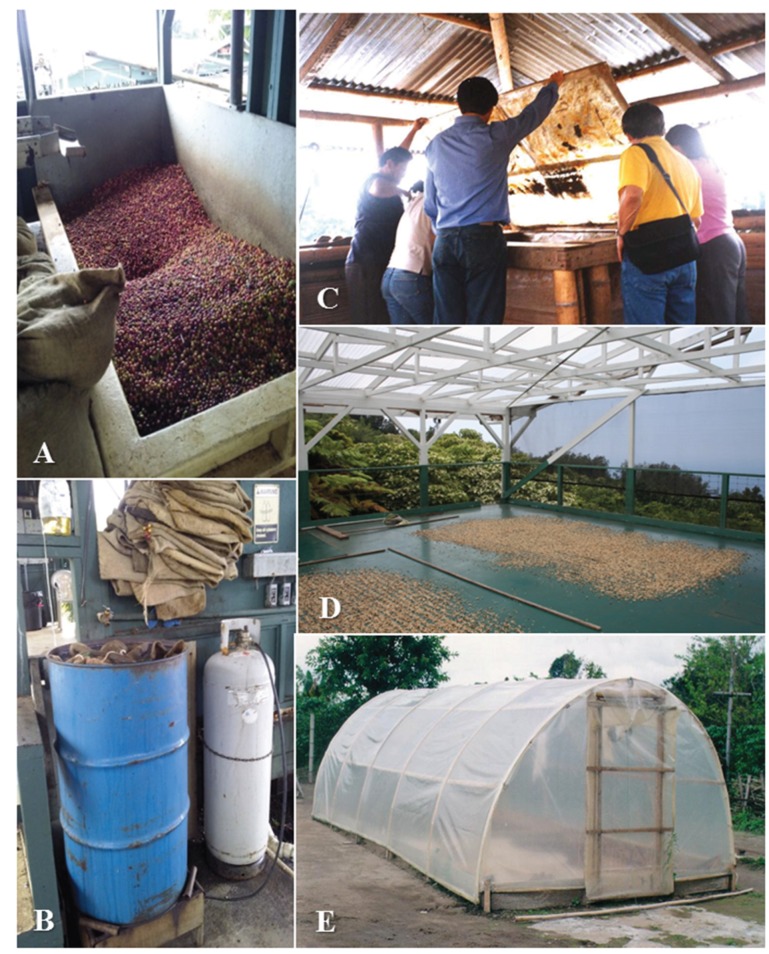
Silos used to process harvested coffee without cover allows escape of CBB (**A**), container with hot water to control CBB in harvest bags (**B**); silo cover with transparent plastic smeared with grease to capture CBB in Colombia (**C**); drying parchment coffee in open areas allow escape of CBB (**D**); and enclosure for dry parchment coffee used to prevent CBB escape in Colombia, (**E**). Coffee plantations in Kona, Hawaii and in Quimbaya, Colombia. Photos taken by Luis F. Aristizábal.

Practical methods to prevent re-infestation of CBB include sanitation measures to reduce its escape from harvesting facilities. Tying burlap or plastic bags used by pickers, moving bags from the field to the wet mill twice per day, screening the wet mill, covering silos and pulp pits with transparent plastic smeared with grease, and drying parchment coffee inside screened greenhouses are useful measures [7,64,125]. CBB-infested berries that are separated post-harvest can be treated with hot water to prevent CBB escape. In Colombia, the lead author conducted participatory research with coffee farmers to improve postharvest control measures that resulted in a significant reduction of CBB escape from processing facilities [64,126]. Alcohol-traps can also be used in processing areas to capture residual CBB. There are opportunities to improve post-harvest control in Hawaii (Figure 5).

### 3.6. Natural Enemies of CBB

The identification of specialist parasitoids and predators of the CBB in its endemic region has been used to establish classical biological control programs. Three African parasitoids were introduced to Latin America against established CBB populations: *Cephalonomia stephanoderis*, and *Prorops nasuta* both (Hymenoptera: Bethylidae), are ecto-parasitoids of CBB larvae and pupae, while *Phymastichus coffea* (Hymenoptera: Eulophidae) is an endo-parasitoid of adults [127,128,129,130,131]. Despite initial difficulties [8,129], *C. stephanoderis* and *P. nasuta* became established several years after their introduction in Brazil [132], Ecuador [133] Guatemala [134], Mexico [135,136], and Colombia [137,138], The parasitoid *P. nasuta* survived cold temperatures, dry seasons, and pressures from insecticide applications against CBB in Brazil for more than 40 years after its introduction in 1929 [132]. In addition, *P. nasuta* proved to be better adapted to Colombia coffee plantations compared with *C. stephanoderis* [137,138,139]. By contrast, *C. stephanoderis* but not *P. nasuta* become established in Mexico [135,136,140].

The level of control achieved in CBB from these exotic parasitoids has been variable. In Ecuador *C. stephanoderis* parasitism rates ranged from 9%–52% [141], in Mexico from 1%–20% [20], and in Colombia 3%–65% [142]. Significant reduction of CBB by *C. stephanoderis* was reported in Mexico [143], and Colombia [144,145,146]. Parasitism rates from *P. nasuta* were low in Brazil (>2%) [147] but higher (27%) in Ecuador [133] and Colombia (50%) [138]. The effectiveness of CBB parasitoids was assessed by the primary author in Colombia through participatory research programs with coffee famers. Significant reduction of CBB populations were obtained over two years in 34 small coffee farms by the combined efforts of releasing *C. stephanoderis* and *P. nasuta* as well as improving the efficacy of harvesting [139,148]. Results showed CBB infestation levels amongst individual farms declined from 7%–48% to 2%–4%, while the production of parchment coffee with <2% damage similarly declined during this period [139]. Since frequent harvesting may affect the establishment of parasitoids by removing infected CBB, the use of screened enclosures has been proposed [148,149,150]. These enclosures have holes that allow the escape of parasitoids but not CBB. Placing CBB-infested berries inside the screened enclosures in the field can be used as a conservation biological control strategy to enhance parasitoids and facilitate their establishment [149,151,152].

Once imported, mass production of parasitoids is needed to improve the chance of establishment. For example, when *P. coffea* was introduced to Colombia from Togo (Africa) a mass production method was developed [131,153], which enabled the species to be transported to other countries in South America as well as India. *Phymastichus coffea* attacks the CBB female before it initiates oviposition thus preventing damage to berries [8,66]. Several studies evaluated the effectiveness and establishment of *P. coffea* in Colombia [66,154,155,156]. High parasitism rates (85%) were initially reported by Jaramillo *et al.* [66]. However, several years after its introduction, *P. coffea* could not be found [115]. In Mexico, *P. coffea* also did not establish [157]. Climate may play an important role in the establishment of this endo-parasitoid.

The USDA-ARS is seeking permits to introduce and test CBB parasitoids in Hawaii under quarantine conditions against potential non targets, including 20 native *Xyleborus* spp. [123]. Some locations that have continuous flowering and berries all year (e.g., Pahala and elevated plantations in Kona) may be suitable for their establishment. In addition, feral coffee plantations, which are CBB reservoirs, also may represent good release sites.

Generalist natural enemies of CBB include ants (*Solenopsis geminate*, *Dorymyrmes* sp. *Pheidole* sp. and *Mycocepurus smithii* in Colombia [158], *Tetramorium bicarinatum* in Cuba [159]), and the predatory thrips *Karnypthrips flaves* in Kenya [160,161]. The generalist predator flat bark beetle, *Cathartus quadricollis* (Coleoptera: Cucujidae), was reported in Colombia [162], Costa Rica [163] and Hawaii [164]. According to Follett [165], currently, *C. quadricollis* is being reared and released in Hawaii by the USDA in Hilo. Additional evaluations are needed to assess the effectiveness of this predator under different field conditions.

Entomopathogenic nematodes which naturally occur in the soil are potential biological control agents for CBB inside fallen berries [65,166,167]. In Colombia field applications of native strains of *Heterorhabditis* sp. (Rhabditida: Heterorhabditidae) and *Steinernema* sp. (Rhabditida: Steinernematidae) applied at high rates/volume (1.25 − 5 × 10^5^ IJ in 300 mL/tree) located, infected and reproduced inside CBB causing mortality of 42% and 34%, respectively, on infested berries on the ground [167]. Lower effectiveness was reported in another study in Hawaii [168] where *S. carpocapsae* caused 4.7% and 17.1% mortality of CBB adults and larvae, respectively, under field conditions. The evaluation of additional native and commercial strains and application strategies for entomopathogenic nematodes in Hawaii is warranted.

### 3.7. Control During “Zoqueo”

In Colombia, coffee lots are pruned back close to the ground every 5 or 6 years, a practice of rejuvenation known as “zoqueo”. While promoting vigorous plants and maintaining manageable sized trees, a major disadvantage of “zoqueo” is that lots are not routinely harvested for one or more years until the coffee production returns to normal. Because CBB management traditionally is not conducted on felled trees (which are not removed), large numbers of CBB may develop on berries and re-infest surrounding areas [8,38]. Measures to control CBB are therefore recommended following “zoqueo”. For example, CBB dispersal can be substantially reduced in “zoqueo” lots through using trap trees, and harvesting and applying *B. bassiana* to these trap trees and ground [169].

**Figure 6:**
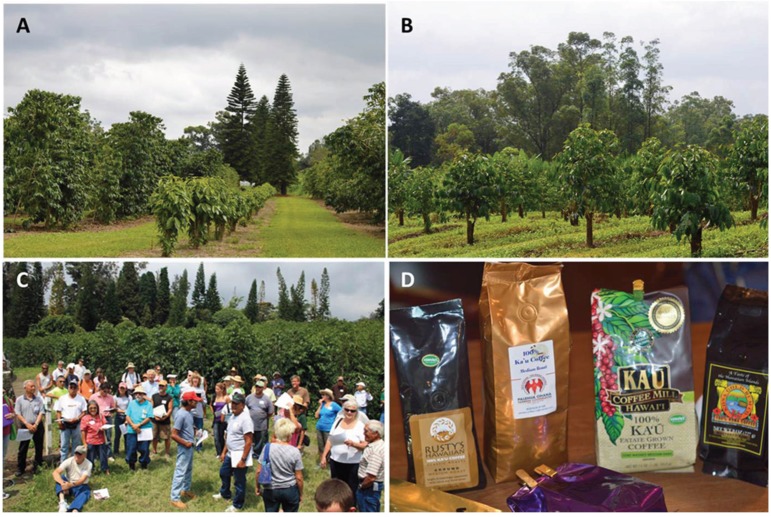
Traditional Kona-style and Beaumont-Fukunaga pruning (**A**); stump pruning by blocks (**B**); training coffee farmers and technicians on IPM (**C**); coffee brands produced in Kau and Kona, Hawaii (**D**). Coffee plantations in Pahala and Kona, Hawaii. Photos taken by Libia C. Mahé and Luis F. Aristizábal.

In Hawaii, rejuvenation practices known as “Kona Style System” and “Beaumont-Fukunaga System” [39,170], allow pruning by rows of mixed age coffee trees to simplify management and harvesting (Figure 6A,B). Those practices need to be re-examined under the presence of CBB, since a combination of old and new coffee trees in the same lots may promote the dispersal of CBB.

## 4. General Recommendations

### 4.1. Recommendation to Coffee Farmers

There is currently no clear strategy to eradicate the CBB once it becomes established. Therefore, coffee farmers need to learn how to produce high quality coffee in the presence of this pest (Figure 6C,D). Recommended strategies include delegating someone to supervise and record all activities related to the control of CBB. In small coffee farms, the owner or family member should assume this role. Applying fertilizers correctly, controlling weeds, pruning verticals, and maintaining irrigation are important to maintain healthy coffee trees. 

The coffee plantation should be divided into management blocks containing coffee lots of similar age. Infestation levels of CBB should ideally be monitored in each coffee lot monthly using the 30-tree method (see Section 3.1). An electronic log of pest infestation levels (including zeros) should be maintained. This will highlight high risk areas (which can receive additional scrutiny and provide good locations to place traps). Any ‘hot spots’ should be identified and targeted for control measures. *B. bassiana* or other pesticide should be applied in coffee lots with >2% infestation and 50% of CBB in an AB position. An approved surfactant will help increase the efficiency of the spray. For rejuvenation of coffee trees, stump pruning by blocks is recommended; since this practice allows control the CBB and delay the re-infestation for 1–2 years (see Section 3.7).

During harvest periods, all over-ripe, and raisin berries should be collected for disposal. Increasing the frequency of harvesting (especially in hot spots) to every 2–3 weeks will help remove CBB infestations. Pickers should be trained to conduct efficient harvesting, and reduce and collect dropped berries. It is recommended to periodically supervise pickers and evaluate their effectiveness at maintaining <5 berries (mature, over-ripe, and raisin) per tree after a harvesting round (see Section 3.3). 

Postharvest recommendations include tying and moving bags to the wet mill as soon as possible after collection. A floatation tank can be used to separate raisin berries which should be treated to kill residual CBB, for example by boiling at 50 °C for 20–30 min. Silos and pulp pits can be covered with a transparent plastic lid smeared with grease to capture emerging CBB. Screening the wet mill and other processing areas and using traps will reduce CBB escape. 

### 4.2. Recommendation to Extension Technicians 

The Extension service plays an important role in the success of establishing IPM for new invasive pests such as the CBB. In Hawaii, many farmers and technicians have insufficient knowledge about the CBB and its control measures, which can promote its spread among and between islands. Adapting control strategies for coffee plantation conditions in Hawaii is needed. For example, the physiology of the coffee plant which drives pest cycles will vary across altitudes and locations. It is important to determine seasonal flight activities in different locations and communicate the optimal periods to apply controls measures such as *B. bassiana* to growers.

The Extension service may provide guidance through Farmer Field Schools. This methodology based on “Learning by doing” allows coffee farmers and workers to understand the CBB problem and practice IPM solutions, and has been successfully implemented in Colombia [76,95,171,172]. Training coffee farmers and pickers is needed. According to grower surveys, cultural harvesting is the IPM component with the highest adoption in Colombia [173,174].

### 4.3. Recommendations to Research Centers

From 1865 articles published on CBB in a recent biography [69], only 42 (<2.2%) included the term “Integrated Pest Management” or “IPM” and only 22 (<1.1%) include the terms “Coffee farmers,” or “coffee growers.” If coffee farmers are expected to adopt IPM techniques for CBB developed at research centers, they need to be included in the development, and validation of these technologies. Some methods may have a higher impact because results are evident. For example, evaluation of CBB female flight activity using alcohol traps, allows insecticides to be timed correctly. Working with growers to create real-time regional maps of CBB abundance (similar to those achieved with other major pests on IPM PIPE http://www.ipmpipe.org/) might be a worthwhile goal. Planting and monitoring trees as “trap crops” near ports or other strategic areas on non-infested islands should be done to delay CBB establishment in those Islands. 

Research should evaluate control measures under local conditions. For example, the effectiveness of *B. bassiana*, entomopathogenic nematodes or other pesticides should be tested in different climatic regions. Cost-benefit analysis of cultural harvesting and other techniques such as vacuum machines to collect fallen berries should be assessed. Improved management of feral plantation should be studied. Research on exotic parasitoids as classical biological control agents and the search for indigenous natural enemies for potential mass rearing, and release against CBB are options to explore. Finally, ecological studies on the CBB in Hawaiian is needed to better understand the pest.

## 5. Conclusions

Establishment of an IPM program for CBB requires knowledge of the biology, ecology, and behavior of the pest and its relationship with the coffee plant and any alternative hosts. Monitoring CBB populations using the 30-tree method and using traps allows early detection of CBB and location of “hot spots”, which can facilitate control decisions. Improvements in the way harvesting and processing is conducted is a big challenge for coffee famers if they want to produce high quality coffees. Harvesting mature, over-ripe, and raisin berries, limiting fallen berries and implementing post-harvest practices is needed. Applications of *B. bassiana* should be applied when the CBB populations are most vulnerable (>2% infestation of CBB and >50% of CBB on AB position). Cooperation between coffee farmers, extension technicians, university researchers, and others is needed to obtain a high adoption and impact on IPM program for CBB.
